# Silver Ions Drive Ordered Self-Assembly Mechanisms and Inherent Properties of Lignin Nanoflowers

**DOI:** 10.3390/polym15173541

**Published:** 2023-08-25

**Authors:** Kai Chen, Encheng Liu, Shengrong Yuan, Baoquan Zhang

**Affiliations:** 1 State Key Laboratory of Chemical Engineering, School of Chemical Engineering and Technology, Tianjin University, Tianjin 300072, China; 2 College or Textile Science and Engineering (International Institute of Silk), Zhejiang Sci-Tech University, Hangzhou 310018, China; 3 Key Laboratory of Green Cleaning Technology & Detergent of Zhejiang Province, Lishui 323000, China; 4 Zhejiang Provincial Innovation Center of Advanced Textile Technology, Shaoxing 312000, China; yuansr@yeah.net

**Keywords:** lignin, chemical modification, self-assembly mechanisms, nanoflowers, emulsifying activity

## Abstract

Designing anisotropic lignin-based particles and promoting the high-value utilization of lignin have nowadays drawn much attention from scientists. However, systematic studies addressing the self-assembly mechanisms of anisotropic lignin-based particles are scarce. In this work, an interaction including the electrostatic forces and chelating forces between lignin and Ag^+^ was regulated via carboxymethylation modification. Subsequently, the aggregation morphology of carboxymethylated lignin in a Ag^+^ solution was observed via SEM. The result showed that a large number of Ag^+^ intercalated into the lignin molecules when the grafting degree of the carboxyl groups increased from 0.17 mmol/g to 0.53 mmol/g, which caused the lignin molecules to gradually transform from disordered blocks to ordered layers. Dynamics research indicated that the adsorption process of Ag^+^ in carboxymethylated lignin conforms to the Pseudo-first-order kinetic model. The saturated adsorption amount of Ag^+^ in the carboxymethylated lignin reached 1981.7 mg/g when the grafting rate of carboxyl groups increased to 0.53 mmol/g, which then fully intercalated into lignin molecules and formed a layered structure. The thermodynamic parameters showed that the thermal adsorption process conforms to the Langmuir model, which indicates that Ag^+^ is monolayer-adsorbed and intercalated into lignin molecules. Meanwhile, the ΔH values are more than 0, which suggests that this adsorption process is a endothermic reaction and that a higher temperature is conducive to an adsorption reaction. Therefore, self-assembly of lignin in a Ag^+^ solution under 70 °C is more conducive to the formation of a nanoflower structure, which is consistent with our experimental result. Finally, pH-responsive Pickering emulsions were successfully prepared using a lignin-based nanoflowers, which demonstrated their potential as a catalytic platform in the interface catalysis field. This work offers a deeper understanding into the formation mechanism of anisotropic lignin-based nanoflowers and hopes to be helpful for designing and preparing anisotropic lignin-based particles.

## 1. Introduction

Today, energy and environmental issues have become two major issues that must be addressed in the development of human society. These concerns have led to the high-value utilization of renewable biomass resources, making them a new research hotspot [1,2,3]. As the second largest biomass after cellulose, lignin is the only renewable aromatic polymer in nature. Currently, over 50 million tons of industrial lignin is produced annually using paper pulp and biological refining in the world [4,5]. However, lignin heterogeneity, including its complex chemical structure and wide molecular weight distribution, results in the uneven properties of lignin when used in chemical and material preparation, which reduce its value-added applications [6]. According to the statistics, only 5% of industrial lignin is used as a concrete water reducer, dye dispersant, adhesive, adsorbent, and negative electrode additive for lead–acid batteries [7,8,9]. The vast majority of lignin is burned as low-level fuel, which will cause great waste of resources and serious environmental pollution [4]. Therefore, reducing the heterogeneity of lignin in terms of its chemical structure and molecular weight is expected to play a key role in promoting the high-value utilization of lignin.

The conversion of lignin with a complex structure and an irregular shape into a nanospherical structure via self-assembly shows great potential in many fields and is considered one of the effective ways to achieve the high-value utilization of lignin [10,11]. Wang et al. prepared monodisperse lignin nanoparticles with controllable sizes via self-assembly and then constructed their short-range ordered structures, successfully preparing a lignin-based photonic material. This photonic material exhibits high saturation structural colors and good humidity-responsive structural color, which could be widely used in fields such as implantable/wearable optical devices, advanced cosmetics, and intelligent food packaging [12]. Sun et al. obtained three low-heterogeneity lignins through a simple two-step dissolution program and prepared three uniform lignin nanospheres (LNS) with different sizes. Research shows that lignin heterogeneity has a significant impact on the green formation of Pb NPs on nanospheres, and the catalytic activity of Pd@LNS could be significantly improved using low molecular weight lignin fractions as raw materials [13]. Lu et al. synthetized tailored lignin nanospheres with specific sizes and tunable structures via a dissolution dialysis method of lignin from corn straw [14]. The results indicated that tailored lignin nanospheres have different UV-blocking abilities and bacterial culture capacities, which opens up new ideas for the high-value utilization of lignin.

Compared with isotropic spherical nanoparticles, anisotropic spherical nanoparticles would have more advantages in terms of amphiphilicity, magnetism, catalytic activity, and optical and electrical properties due to the anisotropy of their shape or chemical composition, which could initiate an incredible revolution in materials science [15]. Specifically, layered nanoflowers, a class of recently developed anisotropic nanoparticles, have attracted much attention in the fields of catalysis and antibacterial agents [16]. This is because of two reasons: On the one hand, this flower structure not only maintains the high specific surface area and good transparency of the two-dimensional structure but also avoids problems such as stacking and aggregation of the two-dimensional structure [17,18]. On the other hand, their unique fold structure can promote efficient dispersion and exposure of metal nanoparticles [19]. However, it is still a challenge to study the transformation of lignin molecules with complex structures and irregular shapes into ordered layered nanoflowers and their formation mechanism.

In this work, carboxymethyl lignin with different grafting ratios was prepared via nucleophilic substitution. The chemical structure, Zeta potential, and water contact angle of carboxymethyl lignin were characterized via nuclear magnetic resonance, dynamic light scattering, and contact angle meter. Subsequently, the effect of carboxylation degree on the aggregation of lignin molecules in AgNO_3_ solution was explored and a layered nanoflower was successfully constructed. The dynamic and thermodynamic models were used to explore the interaction mechanism between lignin molecules and Ag^+^ and revealed the formation mechanism of a layered nanoflower. Finally, the stable Pickering emulsion was prepared with a layered nanoflower as the emulsifier. The development of this work would provide a new method for the preparation of homogeneous anisotropic lignin-based particles, which has important social, economic, and environmental significance for promoting the high-value utilization of lignin.

## 2. Materials and Methods

### 2.1. Materials

The enzymatically hydrolyzed lignin (EHL) extracted from corncobs was supplied by Shengquan Biotechnology Co., Ltd. (Weifang, China). Silver nitrate, sodium hydroxide (NaOH), hydrochloric acid (HCl), and sodium chloroacetate were obtained from Aladdin Chemical Reagent Co., Ltd. (Shanghai, China). All chemicals were used without further purification. The water used in all experiments was purified using deionization.

### 2.2. Carboxymethylation Modification of Lignin

**Scheme 1:**
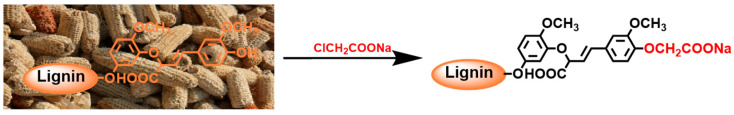
The reaction mechanism of modified lignin.

The carboxylation modification of lignin refers to our previous work [10]. Firstly, 4.0 g EHL and 20 mL 1 mol/L NaOH solution were added into 100 mL three-necked flask and stirred for 1 h at a normal temperature. Then, sodium chloroacetate in different amounts (0.361 g and 1.083 g) was added into above solution, and reaction was stirred for 4 h at 70 °C. The pH value of reaction mixture was adjusted to 7.0 using hydrochloric acid. Subsequently, the reaction mixture was transferred into a dialysis bag (44 mm, 3000 D). Water was changed every 4 h for a duration of 3 days. Finally, carboxymethyl lignin with a black brown solid powder was obtained after concentration and freeze-drying. Meanwhile, the samples were named as EHL-CM-1 and EHL-CM-2. The reaction mechanism of modified lignin is shown in Scheme 1.

### 2.3. Characterization of Carboxymethylated Lignin

The chemical shift of hydrogen atoms in carboxymethylated lignin was characterized using the AVANCE 500 nuclear magnetic resonance spectrometer from Brooke, Switzerland. D_2_O was used as the solvent. The quality of the samples used during the nuclear magnetic measurement process was 100 mg. The group type of the sample was studied using Fourier transform infrared spectroscopy (Thermo Nicolet 5700, Boston, MA, USA). The sample was prepared using potassium bromide compression method. The samples were mixed with potassium bromide in a mass ratio of 1:100 and pressed for 2 min at 8 MPa. Air was used as the background. The test wavelength range for the acquisition was 4000–400 cm^−1^. The contents of carboxylic acid group and phenolic hydroxyl group in carboxymethylated lignin were determined using Metrohm 809 Titrando automatic aqueous phase potentiometric titration instrument of Wantong Company in China (Shanghai, China). Firstly, 30 mg of modified lignin was accurately weighed in a 150 mL beaker and 5 mL of 1 mol/L KOH solution and 50 mg of the internal standard hydroxybenzoic acid were added. Subsequently, 50 mL of deionized water was added to synthesize the modified lignin, and the internal standard was fully dissolved using sonication, and the titration experiment was carried out with HCl as the titration solution to obtain the primary micro-quotient curve. The Zeta potential of samples was measured using the ZUS3100 laser particle size analyzer from Marvin, Hayes, UK, and three measurements were performed at the same concentration. The contact angle of samples was measured using the DSA100 contact angle measuring instrument from Shanghai Klux Scientific Instruments Co., Ltd. (Shanghai, China). Furthermore, the lignin powder was compressed using a tablet press. Then, the contact angle was determined five times in different positions using sessile drop method. The sample powder was pressed into a dense sheet (pressure: 20 MPa, time: 1.5 min) using an infrared tablet press (YP-2 tablet press, Shanghai Shangyue Scientific Instruments Co., Ltd. Shanghai, China). At room temperature and ambient humidity, the contact angle was measured using the static drop method in five different positions.

### 2.4. Aggregation Morphology of Carboxymethylated Lignin in AgNO_3_ Solution

A 15 mL volume of 2 mg/mL carboxymethylated lignin solution was added into 15 mL of 20 mg/mL AgNO_3_ solution and stirred for 5 min at a normal temperature. Then, the above solution was freeze-dried using YTLG-12D freeze-drying machine from Shanghai Yetuo Technology Co., Ltd. (Shanghai, China). Finally, the powder sample was uniformly dispersed on the conductive adhesive and observed using the Gemini SEM 500 scanning electron microscope from Jena in Germany.

### 2.5. Adsorption Kinetics and Thermodynamics of Ag^+^ on Carboxymethylated Lignin

A 15 mL volume of 2 mg/mL carboxymethylated lignin solution was added into 15 mL of 20 mg/mL AgNO_3_ solution. At certain time interval, 50 μL of the above solution was obtained and diluted to 5 mL. The solution was further purified using a 0.22 μm hydrophilic filter head. Finally, the content of Ag^+^ in solution was determined using EXPEC 6500D inductively coupled plasma emission spectrometer (ICP) of China Spectral Education Technology Co., Ltd. (Shanghai, China). The change process of Ag^+^ adsorption rate on carboxymethylated lignin was studied using quasi-first-order kinetic models (1) and quasi-second-order kinetic models (2).

(1) $$\ln\left(Q_{e}-Q_{t}\right)=\ln Q_{e,cal}-K_{1}t$$

(2) $$\frac{t}{Q_{t}}=\frac{1}{K_{2}Q_{e,cal}^{2}}+\frac{t}{Q_{e,cal}}$$

where Q_e_ (mg/g) is the adsorption amount at equilibrium, Q_t_ (mg/g) is the adsorption amount at t min, Q_e,cal_ (mg/g) is the theoretical maximum adsorption amount, K_1_ first-order kinetic rate constant, and K_2_ s-order kinetic rate constant.

Moreover, 15 mL of 2 mg/mL carboxymethylated lignin solution was added into 15 mL of AgNO_3_ solution with different concentrations (10, 15, 20, 25, 30, 35, 40 mg/mL). The above solution was stirred for 10 min at 60 °C, 70 °C, and 80 °C, respectively. Then, the content of Ag^+^ in the solution was determined. Finally, the isothermal adsorption process of Ag^+^ on carboxymethylated lignin was studied using Langmuir model (3) and Freundlich model (4).

(3) $$\frac{1}{Q_{e}}=\frac{1}{Q_{e,cal}}+\frac{1}{bQ_{e,cal}C_{e}}$$

(4) $$\ln Q_{e}=\ln K_{f}+\frac{1}{n}\cdot \ln C_{e}$$

where b (L/g) is the Langmuir constant, K_f_ is the adsorption capacity constant, n is the adsorption strength parameter, and C_e_ (mg/mL) is the Ag^+^ concentration.

Finally, the reaction equilibrium constant with different temperatures and Gibbs free energy are calculated using Van’t Hoff Equation (5) and Gibbs–Helmholtz Equations (6) and (7).

(5) $$\Delta G=-RT\ln K_{c}$$

(6) $$\ln K_{c}=\frac{\Delta S}{R}-\frac{\Delta H}{RT}$$

(7) ΔG=ΔH−TΔS

where R (J/(mol·K)) is the thermodynamic constant, T (K) is the thermodynamic temperature, and K_c_ is the equilibrium constant of Langmuir model adsorption.

(8) $$K_{c}=13,000\times 55.5b$$

### 2.6. Fabrication Process and Performance Analysis of Lignin-Based Layered Nanoflowers

Firstly, the lignin-based layered nanoflowers were prepared by simple regulation of EHL-CM-2 and Ag^+^ concentrations at 70 °C. The formation process of nanoflowers is recorded using SEM. Subsequently, the emulsifying activity of nanoflowers were further studied by referencing our previous work [20]. The lignin-based layered nanoflower-stabilized Pickering emulsion was prepared by applying high-speed shear (PhD Technology LLC, St. Paul, MN, USA) at 10,000 rpm for 5 min. The effects of aqueous phase pH value on the stability and microstructure of Pickering emulsion were investigated. The digital photos and microstructure of the Pickering emulsion were recorded using a digital camera and optical microscope (MV3000, Jiangnan Optical Instrument Factory, Suzhou, China), respectively. The average diameter and size distribution of the droplets were obtained using nanometer software (Nano Measurer 1.2).

## 3. Results and Discussion

### 3.1. Synthesis and Characterization of Carboxymethylated Lignin

Under alkaline condition, carboxymethyl lignin (EHL-CM-1 and EHL-CM-2) with different grafting ratios were prepared by nucleophilic substitution reaction. Table 1 shows that with an increase in sodium chloroacetate feed ratio, the -COOH content in lignin increases from 1.89 mmol/g to 2.42 mmol/g. The grafting degree of carboxyl groups is 0.17 mmol/g and 0.53 mmol/g, respectively. The result shows that the electrostatic interaction between lignin and Ag^+^ will be enhanced after carboxymethylation modification. Meanwhile, with an increase in grafting degree, the weight-average molecular weight of modified lignin increases from 3100 Da to 4200 Da. Interestingly, molecular weight distribution of modified lignin becomes narrower with an increase in molecular weight, and this is the reason for lignin with low molecular weight having higher grafting degree due to its higher reactivity [21]. The above result indicates that carboxymethylation modification could regulate the molecular weight of lignin, which facilitates the preparation of uniform anisotropic lignin-based particles.

**Figure 1:**
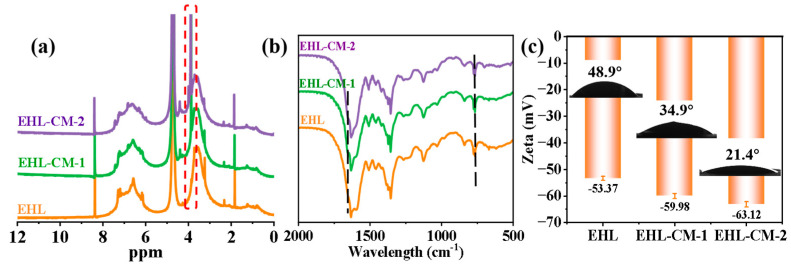
(**a**) ^1^H-NMR, (**b**) FT-IR, and (**c**) Zeta potential and contact angle of EHL, EHL-CM-1, and EHL-CM-2.

As show in Figure 1a, solvent peak of D_2_O appears at δ = 4.80 ppm. The chemical shift of hydrogen in benzene ring appears at δ = 6–8 ppm, which shows that the benzene ring structure in EHL is well preserved. In addition, the chemical shift peak of modified lignin in 3.85 ppm is stronger than EHL. Meanwhile, when the grafting degree of carboxyl groups increases from 0.17 mmol/g to 0.53 mmol/g, the peak intensity of modified lignin also increases, which indicates that sodium chloroacetate is successfully grafted into EHL. Figure 1b shows that the bending vibration peak of benzene ring double substituted C-H and stretching vibration peak of carbonyl groups appears at 768 cm^−1^ and 1725 cm^−1^, respectively. This result further indicates that carboxymethylation modification of lignin is successful. Subsequently, Zeta potential test results show that the negative charge of lignin increases from −53.37 mV to −63.12 mV with an increase in the degree of carboxymethylation (Figure 1c). Meanwhile, lignin hydrophilicity is increased due to the introduction of hydrophilic carboxyl groups.

### 3.2. Aggregation Behavior of Carboxymethylated Lignin in AgNO_3_ Solution

**Figure 2:**
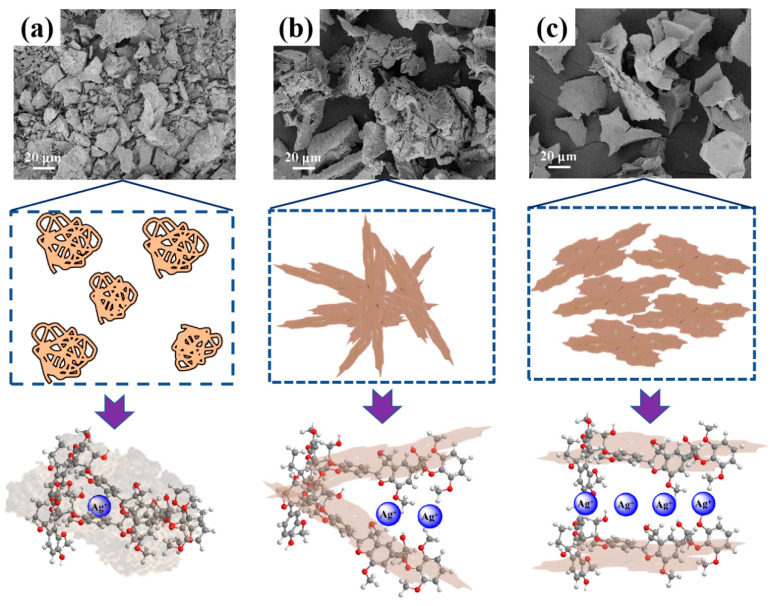
Aggregation morphology and schematic illustration of carboxymethylated lignin: (**a**) EHL, (**b**) EHL-CM-1, and (**c**) EHL-CM-2 in AgNO_3_ solution.

Aggregation morphology of carboxymethylated lignin with different grafting degree in AgNO_3_ solution is shown in Figure 2. Firstly, EHL exhibits severe random aggregation in AgNO_3_ solution due to low carboxyl content and poor hydrophilicity. Aggregation morphology of EHL in AgNO_3_ solution is a massive structure. When the carboxyl group content in lignin increases to 2.06 mmol/g, aggregation morphology of EHL-CM-1 in AgNO_3_ solution is a stacked structure. The reason is that the water solubility of lignin increases due to the introduction of carboxyl groups, which results in the stretching of molecular chains in aqueous solution. Meanwhile, the interaction sites between lignin and Ag^+^ have increased owing to the introduction of carboxyl groups, and more Ag^+^ could intercalate into lignin molecules. When the carboxyl group content in lignin further increases to 2.42 mmol/g, the hydrophilicity of lignin and interaction sites between lignin and Ag^+^ also further increases. Therefore, aggregation morphology of EHL-CM-2 in AgNO_3_ solution displays a single-layered structure. This phenomenon indicates that EHL-CM-2 is expected to self-assemble into layered nanoflowers in AgNO_3_ solution by regulating its concentration. Subsequently, lignin-based layered nanoflowers are successfully prepared by simply regulation of EHL-CM-2 concentration (Figure S1). Firstly, lignin and Ag^+^ form a sheet structure through electrostatic, coordination, and complex interactions when the amount of EHL-CM-2 increases from 0 to 0.01 g (Figure S1a). Then, when the EHL-CM-2 dosage increases to 0.05 g, sheet structure gradually self-assembles into layered nanoflower (Figure S1b). When the EHL-CM-2 concentration further increases, the petal of layered nanoflower close slowly and become a hydrangea (Figure S1c,d). It is suggested that EHL-CM-2 plays a crucial role in the formation of lignin-based layered nanoflowers, which could regulate the morphology of their composites by a simply change in their initial concentration. Meanwhile, high-resolution TEM image reveals that Ag NPs are uniformly dispersed in the petal of the carboxymethyl lignin-Ag-layered nanoflower (Figure S1e).

### 3.3. Adsorption Kinetics of Ag^+^ on Carboxymethylated Lignin

**Figure 3:**
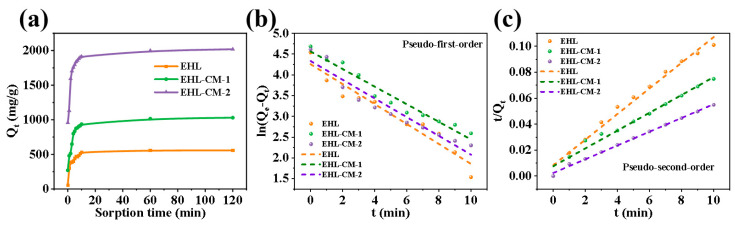
(**a**) Adsorption capacity curves of Ag^+^ in carboxymethylated lignin at different times, (**b**) Pseudo-first-order line of Ag^+^ in carboxymethylated lignin, and (**c**) Pseudo-second-order line of Ag^+^ in carboxymethylated lignin.

As shown in Figure 3a, the adsorption curves of carboxymethylated lignin include three stages: rapid adsorption stage, slow adsorption stage, and adsorption equilibrium stage. When the adsorption time reaches 10 min, the adsorption amount remains basically unchanged and achieves an adsorption equilibrium. Meanwhile, with an increase in the grafting degree of carboxyl groups in lignin from 0 to 0.53 mmol/g, the maximum adsorption capacity of Ag^+^ on lignin increases from 557.8 mg/g to 2018.8 mg/g. This phenomenon indicates that with the introduction of carboxyl groups in lignin, the interaction between lignin and Ag^+^ increases. Subsequently, the effects of different carboxyl groups grafting rates on the adsorption force between Ag^+^ and lignin were further investigated using the Pseudo-first-order kinetic model and Pseudo-second-order kinetic model. As shown in Figure 3b,c and Table S1, the R^2^ of the Pseudo-first-order kinetic model is greater than 98%, which could better simulate the interaction process between lignin and Ag^+^ compared with the Pseudo-first-order kinetic model. However, the three K_2_ value of Pseudo-second-order kinetic model show no significant difference due to the unchanged molecular weight of lignin after modification (Table 1). Interestingly, an increase in the grafting rate of carboxyl groups amplifies the adsorption force between Ag^+^ and lignin. When the grafting rate of carboxyl groups increases to 0.53 mmol/g, the saturated adsorption amount of Ag^+^ in lignin reaches 1981.7 mg/g, and it may fully intercalate into lignin molecules and form a layered structure (Figure 2c).

### 3.4. Adsorption Thermodynamics of Ag^+^ on Carboxymethylated Lignin

The adsorption isotherm model is an important model for studying adsorption phenomena due to the ability to investigate the effect of adsorbate concentration on the adsorption assembly process. Here, the adsorption isotherm of Ag^+^ on EHL-CM-2 is measured at temperatures of 60 °C, 70 °C, and 80 °C, respectively. Meanwhile, the adsorption process of Ag^+^ on EHL-CM-2 is further analyzed using Langmuir model and Freundlich model. As shown in Figure S2 and Table S2, the R^2^ value of the Langmuir model is greater than 0.999, which could better simulate the thermal adsorption process between lignin and Ag^+^ compared to the Freundlich model. Subsequently, the adsorption isotherms of Ag^+^ on EHL and EHL-CM-1 were further studied. Then, the adsorption process was analyzed using the Langmuir model. Figure S3 and Table 2 show that the thermal adsorption process of Ag^+^ on EHL and EHL-CM-1 under different temperature conditions could also be well described. This result indicates that Ag^+^ is monolayer adsorbed and intercalated into lignin molecules, which is consistent with our previous conjecture (Figure 2). As shown in Table 2, with a temperature increase from 60 °C to 80 °C, the maximum monolayer adsorption capacity of Ag^+^ on carboxymethylated lignin and Kc values of Langmuir model also increase. The experimental result shows that this adsorption process is a endothermic reaction, and higher temperature is conducive to adsorption reaction. Meanwhile, the adsorption of Ag^+^ on lignin mainly relies on chemical adsorption. To sum up, self-assembly of lignin in Ag^+^ solution under 70 °C is more conducive to form a nanoflower structure, which is consistent with our experimental result (Figure S1).

Moreover, in order to further analyze the adsorption process, the thermodynamic parameters including Gibbs free energy change (ΔG), enthalpy change (ΔH), and entropy change (ΔS) are calculated. Firstly, based on the Kc value at different temperatures (Table 2), the ΔG value is calculated using the Van’t Hoff equation. As shown in Figure S4 and Table 3, ΔG value for the thermal adsorption process of Ag^+^ in carboxymethylated lignin are less than 0, which indicates that the adsorption process is a spontaneous endothermic process. Subsequently, ΔH and ΔS are further calculated using the Gibbs–Helmholtz equation. Table 3 shows that ΔH value for the thermal adsorption process of Ag^+^ in lignin is greater than 0, which suggests that the adsorption process is a endothermic reaction. Meanwhile, ΔH value of Ag^+^ in carboxymethylated lignin is greater than 20 kJ/mol, which indicates that the adsorption process is mainly a chemical adsorption [22]. This result is consistent with the above data of kinetic and thermodynamic experiments. In addition, ΔS value of Ag^+^ in lignin is greater than 0, which shows that the chaos degree of system increases due to adsorption.

### 3.5. Stability of Lignin-Based Layered Nanoflower-Stabilized Pickering Emulsions

**Figure 4:**
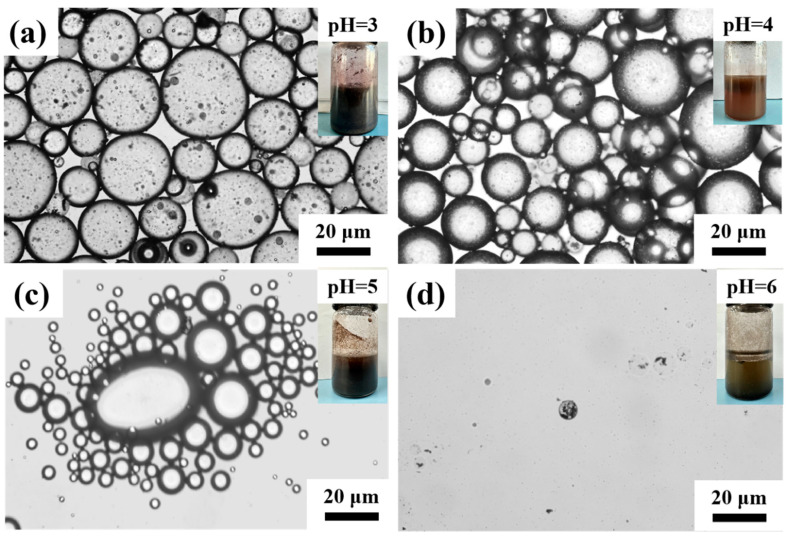
Photographs and optical microscopic images of Pickering emulsions stabilized by 20 mg lignin-based layered nanoflowers in various pH aqueous phases: (**a**) pH = 3; (**b**) pH = 4; (**c**) pH = 5; and (**d**) pH = 6.

In recent years, Pickering emulsions stabilized by solid catalyst particles have been considered as one of the most potential platforms for catalysis due to their uniform oil–water mixing, no diffusion resistance limitation, huge reaction interface, and short molecular diffusion distance [23,24]. Interestingly, switch Pickering emulsion has received special attention in the last 10 years due to its catalyst facile separation and recycling [25]. Therefore, a set of Pickering emulsions were prepared and analyzed in various pH aqueous phases with nanoflowers to build basis for further application. As shown in Figure 4a,b, when the pH value of aqueous phase increases from 3 to 4, the droplet diameter of emulsions decreases from 20 to 10 μm. However, when the pH value of aqueous phase increases to 5, only a portion of the oil phase is stabilized by nanoflowers. When the pH value of aqueous phase further increases to 6, the entire oil phase is not stabilized by nanoflowers. These results indicate that Pickering emulsions stabilized by lignin-based layered nanoflowers have good pH responsiveness, which would have enormous application potential in the interface catalysis field.

## 4. Conclusions

The carboxymethyl lignin with different grafting ratios was successfully synthesized by nucleophilic substitution. Compared with EHL, the carboxyl group content of modified lignin increased by 0.17 mmol/g and 0.53 mmol/g, respectively. The SEM image results showed that the interaction between lignin and Ag^+^ was enhanced with the introduction of the carboxyl group. Therefore, a large number of Ag^+^ ions intercalated into lignin molecules, which caused lignin molecules to gradually transform from disordered blocks to ordered layers. Adsorption kinetics suggested that adsorption process of Ag^+^ in carboxymethylated lignin could be described using the Pseudo-first-order kinetic model. An increase in the grafting rate of carboxyl groups amplifies the adsorption force between Ag^+^ and lignin. When the grafting rate of carboxyl groups increases to 0.53 mmol/g, the saturated adsorption amount of Ag^+^ in lignin reaches 1981.7 mg/g, and it may fully intercalate into lignin molecules and form a layered structure. The thermodynamic parameters for adsorption of Ag^+^ in carboxymethylated lignin show that the thermal adsorption process conforms to Langmuir model, which indicates that Ag^+^ is a monolayer adsorbed and intercalated into lignin molecules. Meanwhile, ΔG value is less than 0, which indicates that the adsorption process is a spontaneous endothermic process. ΔH value is more than 0, which shows that this adsorption process is a endothermic reaction, and higher temperature is conducive to adsorption reaction. Therefore, self-assembly of lignin in Ag^+^ solution under 70 °C is more conducive for the formation of a nanoflower structure, which is consistent with our experimental result (Figure S1). Finally, with the use of natural and biorenewable nanoflowers as Pickering emulsions stabilizers, pH responsive Pickering emulsions were successfully prepared and showed an enormous application potential in the interface catalysis field. This study not only provides a feasible approach to prepare anisotropic lignin-based particles but also offers a deeper understanding on the formation mechanism of anisotropic lignin-based particles.

## Figures and Tables

**Table 1:** Physical and chemical parameters of carboxymethylated lignin.

Compound	Grafting Degree (mmol/g)	*M_w_* (Da)	*M_n_* (Da)	PDI	Ph-OH (mmol/g)	-COOH (mmol/g)
EHL	0	3100	1100	2.82	1.55 ± 0.04	1.89 ± 0.06
EHL-CM-1	0.17	3600	1900	1.89	1.38 ± 0.04	2.06 ± 0.04
EHL-CM-2	0.53	4200	2200	1.90	1.02 ± 0.02	2.42 ± 0.02

**Table 2:** Langmuir model parameters of Ag^+^ in different carboxymethylated lignin.

Temperature (°C)		EHL		EHL-CM-1			EHL-CM-2		
	Q_em_ (mg/g)	K_c_	R^2^	Q_em_ (mg/g)	K_c_	R^2^	Q_em_ (mg/g)	K_c_	R^2^
60 °C	1046.0	73,593	0.9983	1157.5	108,779	0.9996	1234.5	244,588	0.9997
70 °C	1403.3	81,530	0.9316	1464.9	179,089	0.9997	2186.2	561,327	0.9996
80 °C	1811.8	99,567	0.9997	2039.5	287,606	0.9999	3074.9	3,256,851	0.9999

**Table 3:** The thermodynamic parameters for adsorption of Ag^+^ in carboxymethylated lignin.

T (K)	EHL	EHL-CM-1	EHL-CM-2
ΔG (kJ/mol)	−32.3 ± 1.1	−34.5 ± 2.0	−38.7 ± 4.0
ΔH (kJ/mol)	14.7	47.5	125.5
ΔS (J/(mol·K))	173.1	238.8	478.4

## Data Availability

Not applicable.

## Supplementary Materials

The following supporting information can be downloaded at: https://www.mdpi.com/article/10.3390/polym15173541/s1, Figure S1. Aggregation morphology of carboxymethylated lignin with different concentration ((a) 0.01 g, (b) 0.05 g (c) 0.10 g, and (d) 0.20 g) in AgNO_3_ solution at 70 °C, and (e) TEM image of carboxymethyl lignin-Ag-layered nanoflower; Figure S2. (a) Adsorption isotherm of Ag^+^ in EHL-CM-2, (b) Langmuir model of Ag+ in EHL-CM-2, and (c) Freundlich model of Ag^+^ in EHL-CM-2; Figure S3. (a) Adsorption isotherm of Ag^+^ in EHL, (b) Langmuir model of Ag+ in EHL, (c) Adsorption isotherm of Ag^+^ in EHL-CM-1, and (d) Langmuir model of Ag^+^ in EHL-CM-1; Figure S4. The ΔG for adsorption of Ag^+^ in carboxymethylated lignin with an increase of temperature; Table S1. Kinetic model parameters of Ag^+^ in different carboxymethylated lignin solutions; Table S2. Thermodynamic parameters of Ag^+^ in EHL-CM-2 solution.
