# Supplementary material for: Silver Ions Drive Ordered Self-Assembly Mechanisms and Inherent Properties of Lignin Nanoflowers
Source: Polymers (Basel). 2023 Aug 25;15(17):3541. doi: 10.3390/polym15173541 (PMC10489698; doi:10.3390/polym15173541)
Supplement: Supplementary file 1 [file polymers-15-03541-s001.zip › polymers-2547969-supplementary.pdf]

Silver Ions Drive Ordered Self-Assembly Mechanisms and Inherent Properties of Lignin Nanoflowers

Kai Chen [1,2,3,*], Encheng Liu [2], Shengrong Yuan [4] and Baoquan Zhang [1,*]

[1] State Key Laboratory of Chemical Engineering, School of Chemical Engineering and Technology, Tianjin University, Tianjin 300072, China
[2] College or Textile Science and Engineering (International Institute of Silk), Zhejiang Sci-Tech University, Hangzhou 310018, China
[3] Key Laboratory of Green Cleaning Technology & Detergent of Zhejiang Province, Lishui 323000, China
[4] Zhejiang Provincial Innovation Center of Advanced Textile Technology, Shaoxing 312000, China
* Correspondence: chenkai@zstu.edu.cn (K.C.); bqzhang@tju.edu.cn (B.Z.)

Table S1. Kinetic model parameters of Ag^+ in different carboxymethylated lignin solution

Samples	Pseudo-first-order			Pseudo-second-order		
	K_1 (min^{-1})	$Q_{e,cal}$ (mg/g)	R^2	K_2 (g/(mg·min))	$Q_{e,cal}$ (mg/g)	R^2
EHL	0.2404	380.4	0.9382	0.0022	546.4	0.9839
EHL-CM-1	0.2121	673.9	0.9592	0.0009	1021.8	0.9891
EHL-CM-2	0.2254	801.3	0.9277	0.0011	1981.7	0.9971

Table S2. Thermodynamic parameters of Ag^+ in EHL-CM-2 solution

Temperature (℃)	Langmuir				Freundlich		
	b (L/g)	Q_{em} (mg/g)	K_c	R^2	K_f (L/mg)	n	R^2
60	0.339	1234.5	244588	0.9997	713.03	7.522	0.9843
70	0.778	2186.2	561327	0.9996	1666.12	16.113	0.9816
80	4.514	3074.9	3256851	0.9999	2934.05	86.505	0.9807

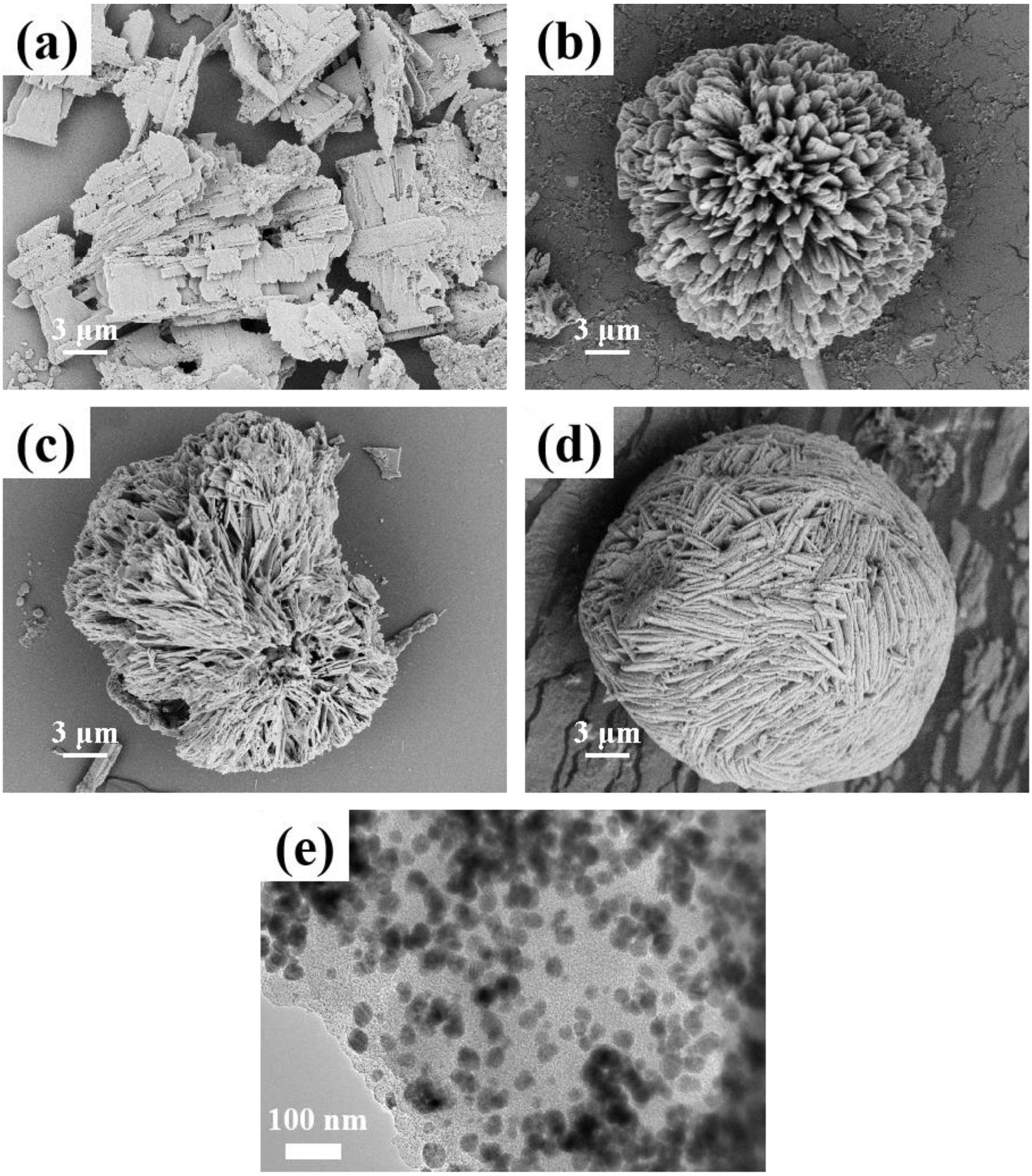

Figure S1. Aggregation morphology of carboxymethylated lignin with different concentration ((a) 0.01 g, (b) 0.05 g (c) 0.10 g and (d) 0.20 g) in $AgNO_3$ solution with 70 ℃, (e) TEM image of carboxymethyl lignin-Ag layered nanoflower

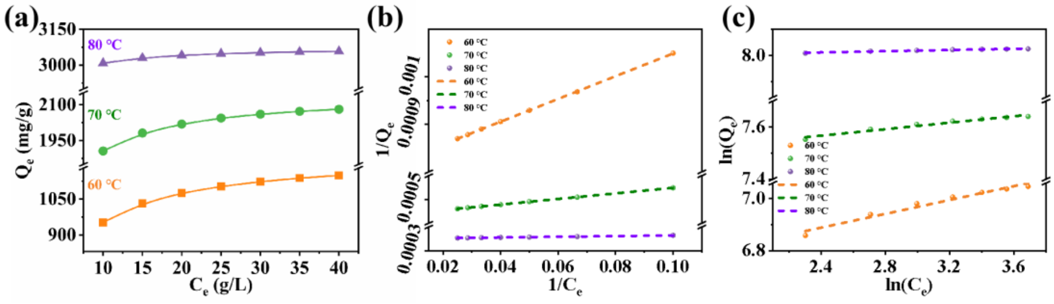

Figure S2. (a) Adsorption isotherm of Ag^+ in EHL-CM-2, (b) Langmuir model of Ag^+ in EHL-CM-2, (c) Freundlich model of Ag^+ in EHL-CM-2

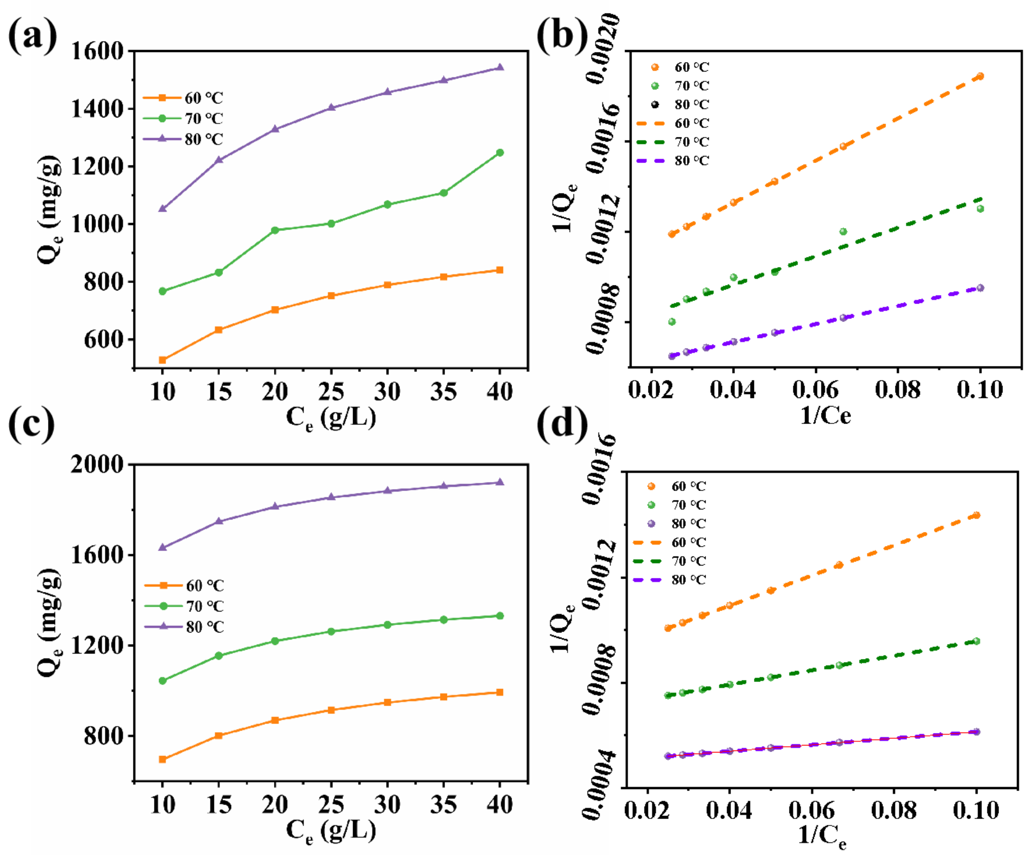

Figure S3. (a) Adsorption isotherm of Ag^{+} in EHL, (b) Langmuir model of Ag^{+} in EHL, (c) Adsorption isotherm of Ag^{+} in EHL-CM-1, (d) Langmuir model of Ag^{+} in EHL-CM-1

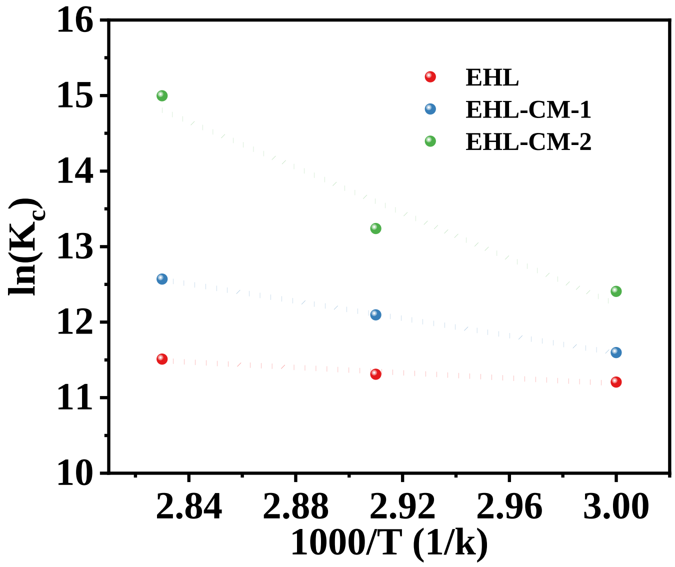

Figure S4. The ΔG for adsorption of Ag^{+} in carboxymethylated lignin with the increase of temperature
